# Plastic deformation of synthetic quartz nanopillars by nanoindentation for multi-scale and multi-level security artefact metrics

**DOI:** 10.1038/s41598-021-95953-0

**Published:** 2021-08-16

**Authors:** Shunya Ito, Toshiyuki Omori, Masao Ando, Hiroyuki Yamazaki, Masaru Nakagawa

**Affiliations:** 1 Institute of Multidisciplinary Research for Advanced Materials (IMRAM), Tohoku University, 2-1-1 Katahira, Aoba-ku, Sendai, Miyagi 980-8577 Japan; 2 Specialty Chemicals Research Center, Shin-Etsu Chemical Co., Ltd., 28-1, Nishifukushima, Kubiki-ku, Joetsu-shi, Niigata, 942-8601 Japan

**Keywords:** Engineering, Materials science, Nanoscience and technology

## Abstract

Individual authentication using artefact metrics has received increasing attention, as greater importance has been placed on the security of individual information. These artefact metrics must satisfy the requirements of individuality, measurement stability, durability, and clone resistance, in addition to possessing unique physical features. In this study, we proposed that nanostructures of synthetic quartz (SQ) deposited on an SQ plate may provide sophisticated artefact metrics if morphological changes could be intentionally introduced into the SQ nanostructures at certain positions. We fabricated SQ nanopillars using a mass-production method (ultraviolet nanoimprint lithography) and investigated their mechanical deformation using nanoindentation with a spheroid diamond tip through a loading and unloading cycle. The SQ nanopillars with an aspect ratio of 1 (i.e., diameters *D* of 100 and 200 nm with corresponding heights *H* of 100 and 200 nm, respectively) could be plastically deformed without collapsing within a specified pillar-array format at programmed positions. The plastically deformed SQ nanopillar arrays demonstrated multi-scale (sub-millimetre, micrometre, and nanometre) and multi-level (shape, area, diameter, and height) individuality authentication and clone resistance. Because SQ is physically and chemically stable and durable, individuality authentication can be a highly reliable tool on Earth and in space.

## Introduction

Individual authentication using artefact metrics has received increasing attention, as a greater emphasis has been placed on the security of individual information^1^. Such artefact metrics utilise physical features unique to individual objects in terms of their optical, magnetic, electric, and/or mechanical properties. For example, ordinary paper has an identifiable surface consisting of microscopic and random patterns of microscale bundles of cellulose nanofibres^2^. Moreover, composite paper containing single-wall carbon nanotubes generates unique photoluminescence images^3^. Magnetic microfibres combined with organic polymer fibres demonstrate an inherent texture arising from their magnetic properties^4^. Integrated circuits with the variations of wires and transistors produce arbiter-based unclonable functions^5^. Randomly collapsed polymer resist nanostructures^6^ and random silicon nanostructures^7^ fabricated using lithographic methods can be identified optically. In addition to having such unique physical features, the artefact metrics must satisfy the following requirements: individuality, measurement stability, durability, and clone resistance. To date, mainly flammable organic artefact metrics have been proposed^2–4, 6^. Physically and chemically stable and durable artefact metrics should comprise inflammable inorganic materials such as mineral sapphire and quartz so that they can be highly reliable media on Earth and in space for multilevel individual authentication.

Synthetic quartz (SQ), also known as synthetic fused silica, exhibits high stability and durability under ambient radiation, light exposure, and temperature in the presence of chemical substances, including acids and organic liquids. SQ possesses a low thermal expansion coefficient and has been utilised as a reliable material in many fields. For example, SQ lenses are essential in optics in the visible frequency region^8^. In addition, SQ plates with selective metallization are used as light-shielding and greyscale photomasks in conventional and advanced photolithography^9^. SQ blocks are used as standard substances in nanoindentation tests^10^. The micro/nanostructures of such SQ materials may be fabricated using semiconductor fabrication technologies. These SQ microstructures may be utilised in macro-lens arrays^11–15^. SQ plates with structures smaller than visible wavelengths demonstrate unique optical functions in waveguide circuits^16^, photonic crystals^17, 18^, and metalenses^19^. Plates with concave/convex nanometre-scale patterns can be fabricated from SQ using electron beam (e-beam) lithography and have been used as templating moulds, which demonstrate high mechanical repeatability and light transparency in ultraviolet nanoimprint lithography for the mass production of semiconductors and optical devices^20–23^. Considering these findings, we proposed that the nanostructures of SQ plates may provide artefact metrics if morphological changes can be intentionally introduced into the SQ nanostructures at specified locations.

To achieve this use of SQ plates, we focused on indentation tests of solid materials at arbitrary positions via loading. In general, nanoindentation with a Berkovich tip^24^ is widely used to measure the Young’s modulus of inorganic and organic substances such as metals, oxides, nitrides, polymers, and biomaterials. The Young’s modulus is determined via calculation from a load–displacement curve recorded using nanoindentation measurements upon loading and unloading until an arbitrary peak of an applied force is achieved, which is generally within the range of micro- to milli-Newtons^24^. Because nanoindentation testing systems are typically equipped with a microscope for determining the position at which the probing tip is pressed downwards, anyone can observe and measure the indentation area optically. In the literature, the Young’s moduli of concave and convex regions of silicon (Si)^25^ and polymer microstructures^26^ have been investigated using nanoindentation with Berkovich diamond tips and plastic deformation of polystyrene micropillars and lines using spherical tips^27, 28^, similar to those used in this study. We derived the idea that by selecting adequate loading forces and positions, deformed shapes exhibiting fine patterns with a specified distribution may be achieved; these patterns may be used to assign individuality and clone resistance to SQ nanostructures.

**Figure 1:**
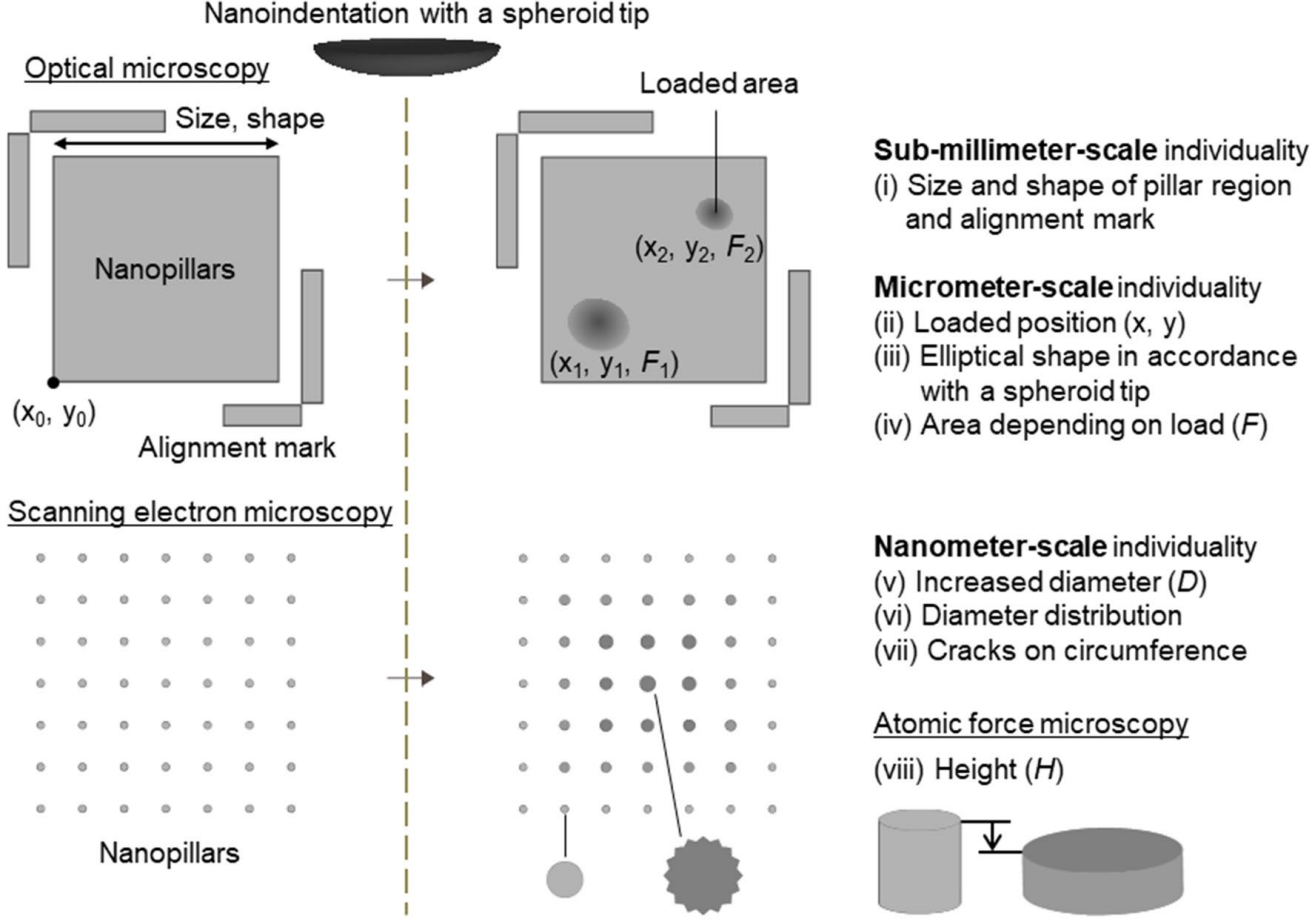
Multi-scale (sub-millimetre, micrometre, and nanometre) and multi-level (shape, area, diameter, and height) individuality authentication and clone resistance of long-term reliable synthetic quartz (SQ) nanopillars after plastic deformation by nanoindentation.

In this study, we fabricated SQ nanopillars using a mass-production method involving ultraviolet nanoimprint lithography and investigated their mechanical deformation via nanoindentation testing with a spheroid diamond tip through a cycle of loading and unloading. The changes in the mechanical shape of the SQ nanopillars with initial diameters of 100 and 200 nm and heights of 100 and 200 nm were studied in terms of their plastic deformation and failure. Additionally, the applicability of plastically deformed SQ nanopillars by nanoindentation is discussed for use in multi-scale and multi-level individuality authentication, as well as resistance, as illustrated in Fig. 1.

## Results

### Mechanical collapse and plastic deformation

**Figure 2:**
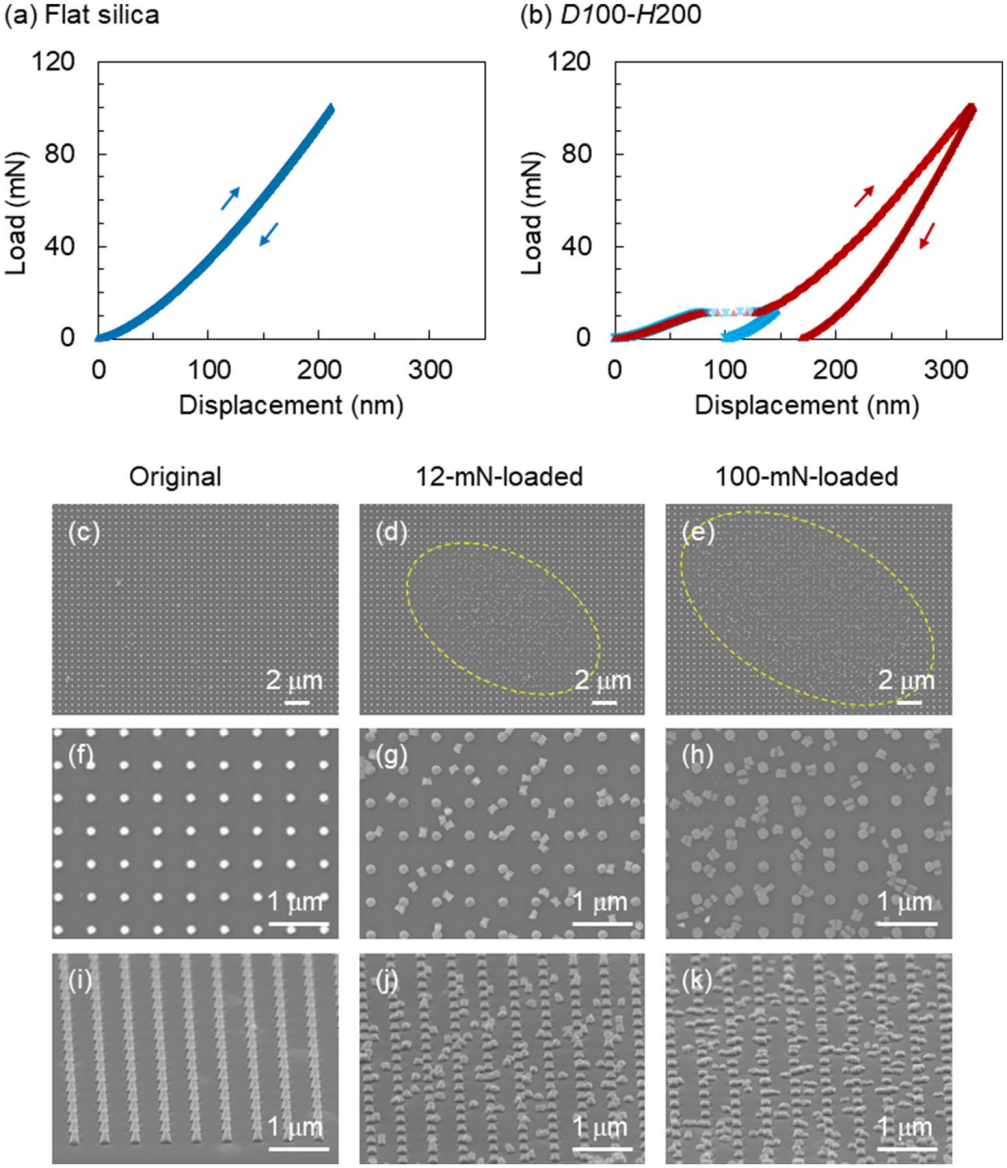
(**a**,**b**) Load–displacement curves of SQ with (**a**) a flat surface until a peak load of 100 mN and (**b**) *D*100–*H*200 nanopillars until a peak load of 12 mN (blue plots) and 100 mN (red plots) measured by nanoindentation. (**c**–**h**) Surface and (**i**–**k**) tilted SEM images of the (**c**,**f**,**i**) original *D*100–*H*100 SQ nanopillars and those after deformation via nanoindentation until a peak load of (**d**,**g**,**j**) 12 mN and (**e**,**h**,**k**) 100 mN.

First, we compared the morphological changes of a flat SQ plate and an SQ plate with SQ nanopillars of *D*100–*H*200 (i.e., a diameter *D* of 100 nm and height *H* of 200 nm) with an aspect ratio (*H*/*D*) of 2 by nanoindentation testing with a spheroid diamond tip. The SQ plate (Fig. 2a) with a thickness of 0.6 mm underwent elastic deformation without mechanical hysteresis between the loading and unloading curves until reaching a peak load of 100 mN. This elastic deformation using the spheroid diamond tip differed from a previously reported plastic deformation of an SQ plate with mechanical hysteresis using a Berkovich diamond tip with a sharpened three-sided pyramid shape^24^. The elastic deformation of the flat SQ plate enabled us to study the mechanical deformation of SQ nanopillars on the SQ plate. Figure 2b illustrates the load–displacement curves of the *D*100–*H*200 SQ nanopillars measured using a spheroid diamond tip. A loading/unloading hysteresis appeared in the load–displacement curve (red plots) until a peak load of 100 mN. During the progression to the peak load, a plateau region appeared at approximately 10 mN in the displacement range of 70–140 nm. To confirm the occurrence of mechanical hysteresis, the load–displacement curve (blue plots) was recorded until a peak load of 12 mN. The presence of the plateau region indicated the destructive fracture deformation of the SQ nanopillars during the plateau in the load–displacement curve.

To identify deformation of the *D*100–*H*200 SQ nanopillars, we compared the field emission scanning electron microscopy (FE-SEM) images of the original SQ nanopillars before (Fig. 2c,f,i) and after loading until peak loads of 12 mN (Fig. 2d,g,j) and 100 mN (Fig. 2e,h,k). As depicted in Fig. 2d,e, the low-magnification surface images confirmed the presence of a discernible elliptical shape at the probed position upon indentation testing. The elliptical shape and the direction of its long axis, which were in accordance with the shape of the spheroid probing tip, could also be visualised by optical microscopy (Supplementary Fig. S1, Supporting Information). A comparison between Fig. 2d,e revealed that the area of the elliptical shape at a peak force of 100 mN was larger than that at a peak force of 12 mN. Therefore, the area of the elliptical shape depended not only on the shape of the spheroid probing tip but also on the applied peak load. This area enlarged as the applied peak load was increased. The high-magnification surface images (Fig. 2f–h) and tilted images (Fig. 2i–k) confirmed that the *D*100–*H*200 SQ nanopillars collapsed in the plateau region at approximately 10 mN, and fractures were randomly distributed. Interestingly, the peak load of 12 mN caused the *D*100–*H*200 SQ nanopillars to fracture in the in-plane direction parallel to the SQ substrate surface and near the middle of the height of the nanopillars. Tilted FE-SEM images revealed that the pillar height of 207 ± 5 nm decreased to 90 ± 8 nm after applying a peak load of 12 mN. The ellipse-shaped area of the underlying SQ substrate, where approximately 480 SQ nanopillars were fractured, was 1.6 × 10^2^ µm^2^ using FE-SEM (Fig. 2d). Under the assumption that the 480 SQ nanopillars with a top diameter of 100 nm (and a top area of 7.85 × 10^3^ nm^2^) had an approximate contact area of 3.8 µm^2^, the peak load of 12 mN corresponded to a pressure of 3.2 GPa. Applying a peak load of 100 mN not only resulted in further fracturing of broken species distributed randomly on the substrate surface but also resulted in the plastic deformation of residual stubs remaining on the substrate surface, as depicted in Fig. 2h,k. Several broken pieces were split in half in the out-of-plane direction along the pillar height. The FE-SEM observations demonstrated that the *D*100–*H*200 SQ nanopillars with an aspect ratio of 2 were fractured near the centre of the pillar height at 12 mN. Subsequently, the residual stubs on the SQ substrate surface caused plastic deformation at 100 mN.

### Plastic deformation of low-aspect-ratio nanopillars

**Figure 3:**
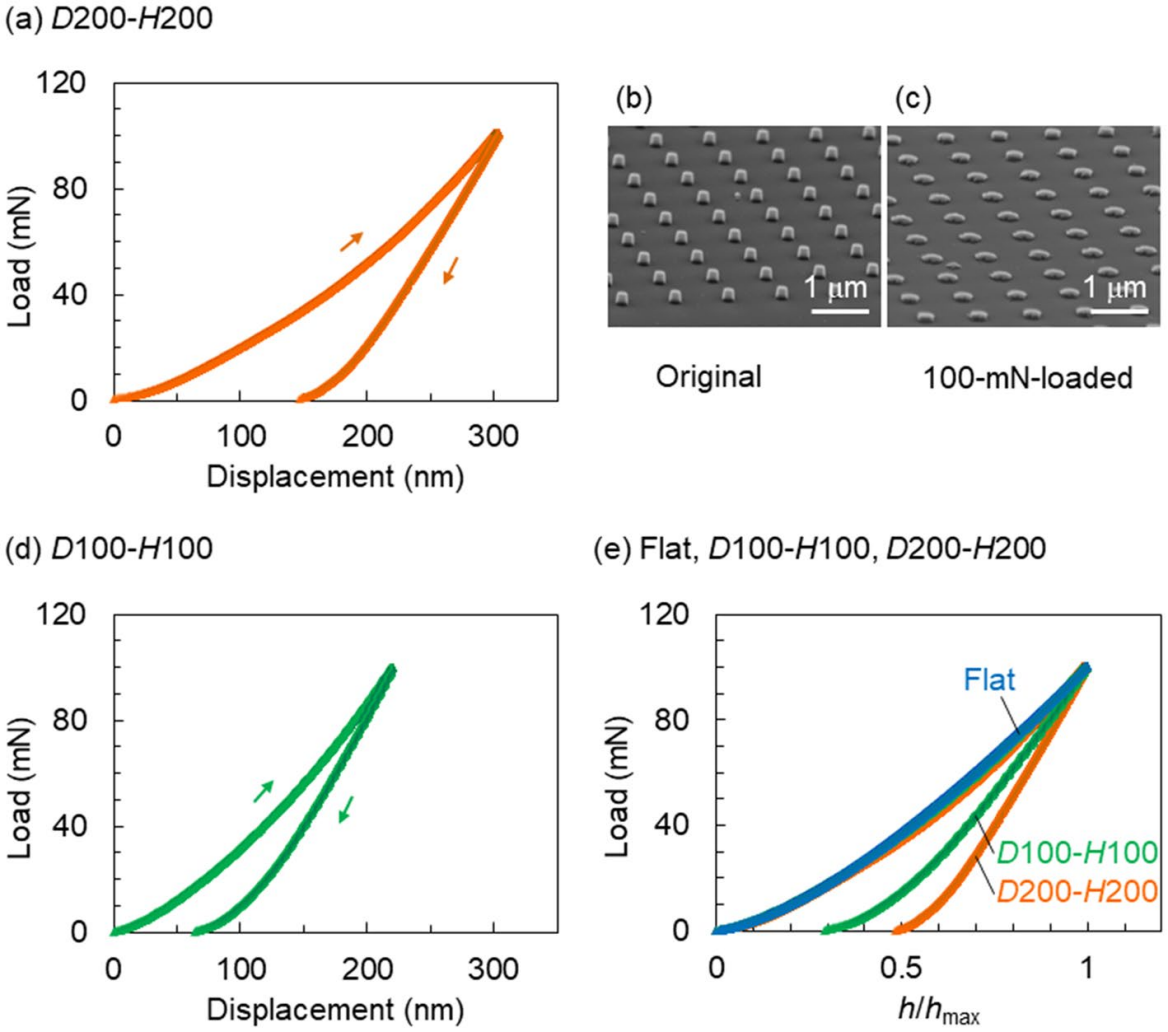
Load–displacement curves of the (**a**) *D*200–*H*200 and (**d**) *D*100–*H*100 SQ nanopillar surfaces. (**b**,**c**) Tilted SEM images of the (**b**) original *D*200–*H*200 SQ nanopillars and (**c**) the pillars after deformation at a peak load of 100 mN. (**e**) Relationship between the normalised displacements (*h*/*h*_max_) and loads measured for SQ with a flat surface, as well as with the *D*100–*H*100 and *D*200*–H*200 nanopillars.

Figure 3a,d depict the load–displacement curves of the *D*200–*H*200 and *D*100*–H*100 SQ nanopillars, respectively, with a low *H*/*D* value of 1 measured at a peak load of 100 mN. The *D*200–*H*200 SQ nanopillars demonstrated a larger loading/unloading hysteresis of 150 nm than the *D*100–*H*100 SQ nanopillars with a hysteresis of 70 nm. The absence of a plateau region in these load–displacement curves upon loading indicated the plastic deformation of the *D*200–*H*200 and *D*100*–H*100 SQ nanopillars without fracture deformation. To confirm this plastic deformation, we compared the FE-SEM images of the original *D*200–*H*200 SQ nanopillars (Fig. 3b) and those mechanically deformed at a peak load of 100 mN (Fig. 3c). No fractures were observed in the latter. The initial diameter of 200 nm was enlarged concomitantly with the decrease in the initial height of 200 nm. As depicted in Fig. 3b,c, the loading process caused plastic deformation of the *D*100*–H*100 nanopillars until a load of 100 mN was reached, wherein the initial diameter enlarged and fractures were not observed. It was confirmed that nanoindentation of the *D*100*–H*100 and *D*200–*H*200 SQ nanopillars with an aspect ratio of 1 using a spheroid tip resulted in plastic deformation without fractures.

To investigate the differences between the plastic deformations of the *D*100*–H*100 and *D*200–*H*200 SQ nanopillars, the load–displacement curves were compared in detail. The maximum displacement (*h*_max_) values at a peak load of 100 mN were 220 nm for *D*100*–H*100 and 300 nm for *D*200–*H*200. The *h*_max_ values were larger than the respective pillar heights. This result indicates that until the peak load was reached, the nanoindentation induced plastic deformation of the SQ nanopillars together with elastic deformation of the underlying SQ plate surface. The displacement values after the unloading reached 0 mN were 70 nm for *D*100–*H*100 and 150 nm for *D*200–*H*200. Figure 3e depicts the load–displacement curves replotted as a function of *h*/*h*_max_ to normalise the practical displacement (*h*) with the maximum displacement (*h*_max_). Until the peak load was reached, the load–displacement curves of the *D*100*–H*100 and *D*200–*H*200 SQ nanopillars were almost identical to that of the flat SQ plate without nanopillars. After unloading, the *D*200–*H*200 SQ nanopillars exhibited a higher *h*/*h*_max_ value than the *D*100–*H*100 SQ nanopillars. This finding suggests that the *D*200–*H*200 SQ nanopillars induced larger plastic deformation than the *D*100–*H*100 SQ nanopillars regardless of the same aspect ratio of 1.

**Figure 4:**
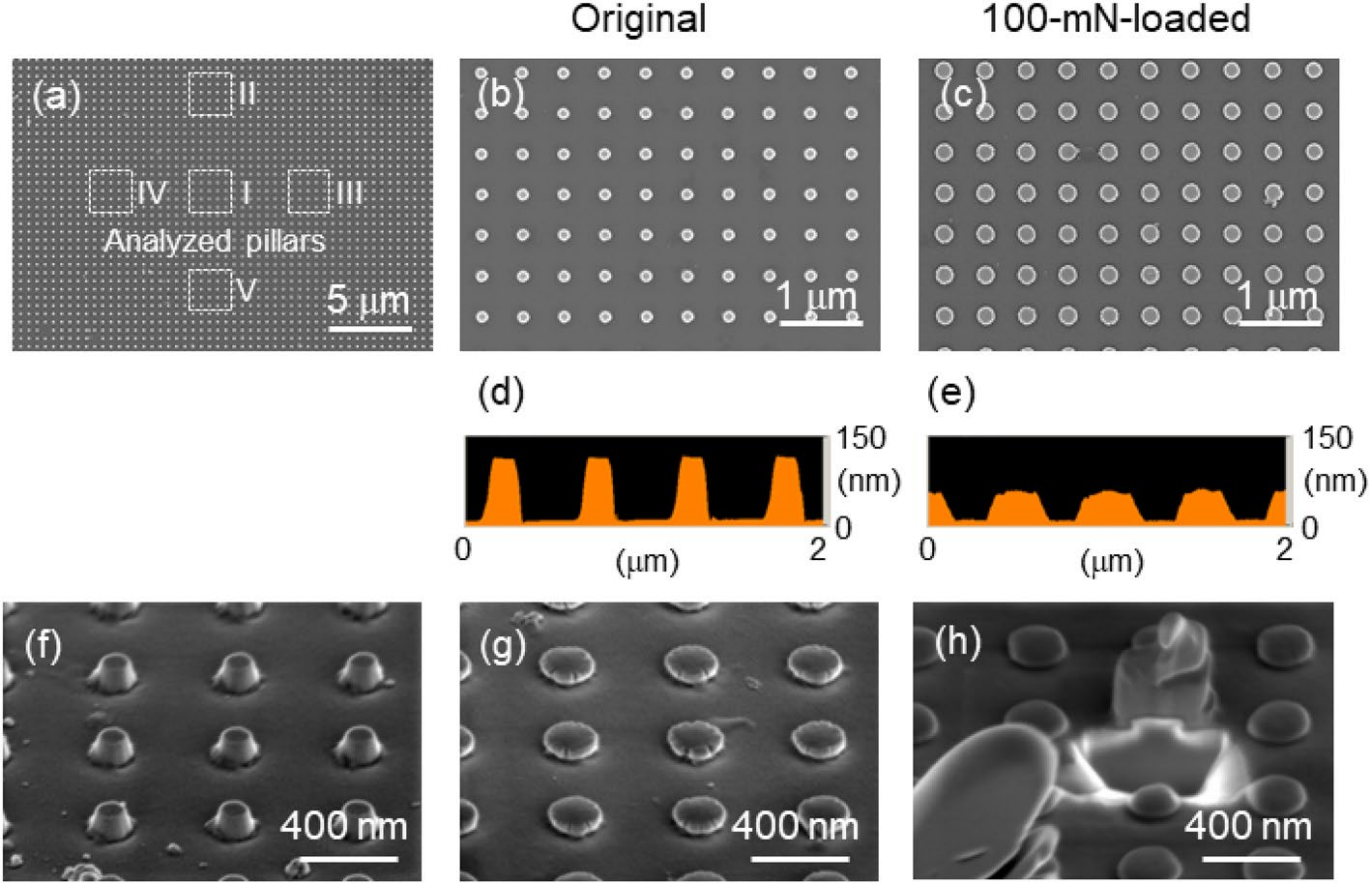
(**a**–**c**) Surface SEM images and (**d**,**e**) AFM height profiles of the (**b**,**d**) original *D*100–*H*100 SQ nanopillars and (**a**,**c**,**e**) the pillars after deformation at a peak load of 100 mN. (**f**,**g**,**h**) Tilted SEM images of the (**f**) original *D*100–*H*100 SQ nanopillars and (**g**) those after deformation and (**h**) after deformation and exposure to an FIB to observe the cross-sectional image of a deformed SQ nanopillar.

Based on the contrasting brightness of the transformed *D*100*–H*100 SQ nanopillars, FE-SEM imaging revealed an elliptical indentation shape with long- and short-axis lengths of 17 and 10 µm, respectively, which were discernible at the position at which the spheroid diamond tip was pressed, as depicted in Fig. 4a. The elliptical indentation shape had an approximate area of 170 µm^2^, which was reflected by the individual shape of the spheroid tip. A morphological analysis of 25 transformed SQ pillars present in five square regions labelled I–V within the elliptical indentation shape was conducted in terms of their average diameter (*D*_ave_), maximum diameter (*D*_max_), and minimum diameter (*D*_min_) determined via FE-SEM, and the results are summarised in Table 1. The average diameters of the transformed SQ nanopillars were 179 (region I), 127 (region II), 143 (region III), 114 (region IV), and 130 (region V) nm.

**Table 1:** Analysed regions I–V (Fig. 4a) and characterised diameters (*D*_ave_, *D*_max_, and *D*_min_) after the deformation of the *D*100–*H*100 SQ nanopillars.

Region	I	II	III	IV	V
*D*_ave_	179	127	143	114	130
*D*_max_	188	136	158	134	138
*D*_min_	172	118	128	100	120

To clarify the shape changes in the *D*100–*H*100 SQ nanopillars achieved by loading until 100 mN, the diameter and height were determined by FE-SEM and atomic force microscopy (AFM) and compared before and after loading. Before nanoindentation, the *D*100–*H*100 SQ nanopillars had an average top diameter of 105 ± 3 nm (Fig. 4b) and height of 105 nm (Fig. 4d) with a truncated cone shape, as illustrated in the tilted FE-SEM image (Fig. 4f). Prior to reaching a peak load of 100 mN, nanoindentation transformed the truncated cone shapes to mushroom-like shapes, as depicted in Fig. 4g. The transformed *D*100–*H*100 SQ nanopillars had an average diameter of 228 ± 13 nm (Fig. 4c) and height of 50 nm (Fig. 4e), which were approximately two times larger and half those of the *D*100–*H*100 SQ nanopillars before nanoindentation, respectively. The increase in diameter and decrease in height suggest that loading by nanoindentation caused the transformation of the truncated cone-shaped SQ nanopillars to mushroom-shaped SQ nanopillars via plastic deformation as the sidewalls of the SQ nanopillars spread to peripheral spaces. As depicted in Fig. 4g, random radial cracks were generated along the circumferences of the transformed mushroom-like SQ nanopillars. The focused ion beam (FIB)-SEM cross-sectional image (Fig. 4h) confirmed that the transformed SQ nanopillars possessed a mushroom shape.

The plastic deformation of the *D*100–*H*100 SQ nanopillars from the truncated cones occurred without collapse and was similar to that of the *D*200–*H*200 SQ nanopillars (Fig. 3c). The FIB-SEM image of the mushroom-like *D*100–*H*100 SQ nanopillars after nanoindentation revealed that the bottom sections were barely transformed, whereas the middle and top sections were enlarged and spread to peripheral spaces during plastic deformation. The plastic deformation induced by increasing the load until 100 mN is discussed as follows. The spheroid diamond tip pressed the tops of the SQ nanopillars inwards. The middle of the SQ nanopillars preferentially spread to their peripheral spaces as the load was increased. The bottoms were barely affected owing to the presence of the underlying SQ substrate. As depicted in Fig. 3e, the *D*200–*H*200 SQ nanopillars exhibited a larger deformation ratio (*h*/*h*_max_ = 0.49) than the *D*100–*H*100 SQ nanopillars (*h*/*h*_max_ = 0.28) after unloading. This result indicates that the peak load of 100 mN caused the tops of the *D*100–*H*100 SQ nanopillars to be displaced by approximately 220 nm due to the transformation of the SQ nanopillars together with the underlying substrate. The surface position of the underlying elastic substrate recovered to its initial state by unloading; however, the initial 100-nm height of the nanopillars decreased by approximately 60 nm to a residual height of 40 nm due to plastic deformation (Figs. 3d, 4e). Considering the *D*200–*H*200 SQ nanopillars, the initial nanopillar height of 200 nm decreased by 150 nm to a residual height of 50 nm (Fig. 3a). Therefore, these nanopillars exhibited a larger plastic deformation than the *D*100–*H*100 SQ nanopillars, although they possessed the same aspect ratio of 1. The load–displacement curve measured for the *D*200–*H*100 SQ nanopillars was almost consistent with that measured for the *D*100–*H*100 SQ nanopillars. The initial height of the *D*200–*H*100 SQ nanopillars decreased by 60 nm after unloading (Supplementary Fig. S2, Supporting Information). These findings revealed that the residual heights obtained after plastic deformation were independent of the aspect ratio of the height to the diameter and were dependent on the height of the nanopillars. The nearly constant residual layer thicknesses of approximately 50 nm strongly suggested the presence of a region that is difficult to transform near the bottom of the SQ nanopillars.

## Discussion

In this section, multi-scale individuality and clone resistance for artefact metrics are discussed based on the plastic deformation of SQ nanopillars. SQ, which is also known as synthetic fused silica, is an exceptionally reliable material considering its chemical and physical stability. As illustrated in Fig. 1, SQ nanopillar arrays on an SQ substrate demonstrate multi-scale individualities at (i) sub-millimetre, (ii)–(iv) micrometre, and (v)–(vii) nanometre scales. The divisions noted in Roman numerals are used to discuss the various properties of these nanopillars. At the sub-millimetre scale, (i) the sizes and shapes of the nanopillar arrays and the positioning alignment marks are discernible using optical microscopy in addition to FE-SEM. (ii) The loaded positions (x, y), (iii) ellipsoidal indentation shape in accordance with the shape of the spheroid diamond tip used for nanoindentation, and (iv) indentation area, which is dependent on the load *F*, can be observed on the micrometre scale using FE-SEM. At the nanometre scale, (v) the diameter *D* and (vi) diameter distribution within the indentation, as well as (vii) the numbers of cracks on the circumferences of the mushroom-like nanopillars, can be discerned using FE-SEM. Additionally, (viii) the heights *H* of the initial and transformed nanopillars can be measured using AFM.

In terms of (i), the sizes and shapes of the nanopillar arrays, as well as the positioning alignment marks, are freely designed and fabricated using e-beam and nanoimprint lithography to achieve array sizes of 100 × 100 µm^2^ and alignment-mark bar sizes of 10 µm in width and 50 or 100 µm in length in this study (Supplementary Figs. S1 and S3h, Supporting Information). The loaded position (ii) is freely determined using an indentation testing apparatus. Because the spheroid diamond tip has an ellipsoidal shape, the ellipsoidal indentation shape (iii) formed on the pillar array reflects this spheroid diamond shape and can be analysed using the long- and short-axis lengths and directions. An increase in the peak load *F* causes the ellipsoidal indentation area (iv) to become enlarged. The plastic deformation of the *D*100–*H*100 SQ nanopillars with an identical diameter and height resulted in an increase in their diameters (v) and a decrease in their heights (viii) with specific distributions (vi) within the indentation. The *D*100–*H*100 SQ nanopillars with an initial truncated cone structure were transformed into mushroom-like structures with cracks (vii) around their spheres. The numbers and positions of these cracks differed among the deformed SQ nanopillars, as depicted in Fig. 4g. Therefore, SQ nanopillars with aspect ratios of approximately 1 could be deformed plastically without failure using nanoindentation with a spheroid diamond tip. Plastically deformed SQ nanopillars can provide multi-scale and multi-level individualities and clone resistances for artefact metrics. However, certifying the individualities of SQ nanopillars with a high aspect ratio, such as the *D*100–*H*200 SQ nanopillars, is difficult because fractures generated by collapse would not be present during use, which would prevent software-based analysis of the diameters of the transformed SQ nanopillars within a specified area. The generated fractures reduce the accuracy of individuality authentication for clone resistance. Because SQ can endure strong deep ultraviolet light and radiation exposure, as well as heat treatment above 1000 °C and chemical treatments with strong acids and common solvents, SQ nanopillars on SQ substrates can be considered to be a highly reliable media for multi-level individual authentication for artefact metrics both on Earth and in space.

In this study, we described multi-scale and multi-level individuality authentication and clone resistance for artefact metrics using SQ nanopillars deformed by nanoindentation. The SQ nanopillars were fabricated using ultraviolet nanoimprint lithography, which is an industrially accepted process that can be conducted at a high throughput and low cost. The SQ imprint moulds were precisely prepared using e-beam lithography. After nanoindentation with a spheroid diamond tip, the SQ nanopillars demonstrated the ability to be deformed plastically without collapsing in a certain pillar-array structure at programmed positions. Mushroom-like SQ nanopillars with peripheral cracks were deformed from SQ nanopillars with a truncated cone structure, and they appeared to possess shapes akin to an island in the ocean. The scale is 1/10^12^. A pillar-top area of approximately 100 × 100 nm^2^ in a pillar-array area of 100 × 100 µm^2^ corresponds to the surface area of the island of Guam (5.5 × 10^2^ km^2^) in comparison with the surface area of the Earth (5.1 × 10^8^ km^2^). Scanning using FE-SEM and AFM resembles observation using an artificial satellite. The pillar-top area was transformed via nanoindentation with a spheroid diamond tip. To identify the shapes of the individual SQ nanopillars and their assemblages, multi-scale observations and multi-level measurement methods, including optical microscopy, FE-SEM, and AFM, are required, as were conducted in this study. Because SQ is physically and chemically stable as well as durable, individuality authentication using plastically deformed SQ nanopillars is expected to be feasible both on Earth and in space.

## Methods

### Fabrication of nanopillars

SQ nanopillars with diameters (*D*) of 100 and 200 nm and heights (*H*) of 100 and 200 nm, abbreviated as *D*100–*H*100, *D*100–*H*200, *D*200–*H*100, and *D*200–*H*200, were fabricated using mass-replicable ultraviolet nanoimprint lithography (UV-NIL). Silica imprint moulds with square-grid hole arrays were prepared by e-beam lithography according to our previous reports^29, 30^. An 80-nm-thick film of a positive-tone resist was prepared by spincoating ZEP520A (ZEON) onto an SQ substrate (Shin-Etsu) with dimensions of 20 × 20 × 0.5 mm^3^ and annealing at 180 °C for 10 min. A top-coating of a conductive organic layer of ESPACER (Showa Denko) was then applied at a spinning speed of 2000 rpm for 60 s, followed by annealing at 70 °C for 3 min in ambient air.

Electron beam drawing was conducted using an ELS-G125S writer (Elionix) at an acceleration voltage of 130 kV and beam current of 100 pA. Square-grid hole arrays (with a pitch of 500 nm for *D*100 and 1000 nm for *D*200) and micrometre-scale bars (with a width of 10 µm and length of 50 or 100 µm) were drawn on separated areas of 100 × 100 µm^2^ grids and around the corners of each hole-array area, respectively. The micron-bars were used as indicators that were discernible using an ENT-2100-equipped optical microscope (Elionix). Resist masks were prepared by being rinsed with ultrapure water (18.2 MΩ cm), immersed in a ZED-N50 developer (ZEON), and rinsed with a ZMD-B solvent (ZEON). The silica surfaces were excavated via electron cyclotron resonance (ECR) etching using an EIS-200ER etcher (Elionix) with C_3_F_8_ gas for 90 s. The residual resist materials were removed by exposure to vacuum UV light using a UEM20-172 Xe excimer lamp (Ushio). Silica imprint moulds separated into 10 × 10 mm^2^ pieces were cleaned by immersion in sulfuric acid and rinsed using ultrapure water and UV/ozone exposure for 20 s using a UVE-110-1H cleaner (Sen Light) before use. The mould surfaces were modified with an antisticking molecular layer formed using tridecafluoro-1,1,2,2-tetrahydrooctyltrimethoxysilane (FAS13) by chemical vapour surface modification at 150 °C for 1 h^31–33^.

UV-NIL was performed as illustrated in Supplementary Fig. S3 (Supporting Information). A UV-curable resin of NL-KK1^34, 35^ was diluted with 1-methoxy-2-propanol and spincoated onto a 4-inch SQ wafer (Shin-Etsu) with a 10-nm-thick CrN hard mask and annealed at 50 °C for 2 min. The modified silica imprint mould and resin-coated SQ wafer were set as a superstrate and substrate, respectively, in an ImpFlex Essential nanoimprint stepper (Sanmei). UV nanoimprinting used for copying the pillar resist patterns was conducted under the following conditions: an applied pressure of 1.0 MPa, 365-nm light intensity at 100 mW cm^−2^, and an exposure duration of 20 s. UV exposure was conducted using an L11921-400 UV-LED (Hamamatsu Photonics). A subsequent three-type dry etching procedure for the selective removal of the residual resist layer, CrN hard mask, and silica was performed. Anisotropic oxygen reactive ion etching (O_2_ RIE) was conducted using a lab-made apparatus^35^ to remove the residual resist layer (conditions: an oxygen-gas mass flow rate of 10 sccm, chamber pressure of 0.5 Pa, and radio frequency bias power of 20 W). Argon ion beam milling using a 20IBE-C apparatus (Hakuto) was then conducted to remove the CrN hard mask (conditions: a beam bias of 600 V, beam current of 400 mA, acceleration voltage of 200 V, and milling duration for 0.5 min). Finally, ECR etching in the same manner as described for the preparation of the silica imprint moulds was used to excavate silica. SQ nanopillars with heights of 100 and 200 nm were prepared via ECR etching for 90 and 180 s, respectively. The SQ wafer was diced into plates (15 × 15 mm^2^), and plates with nanopillar surfaces were fabricated using stripping masks via exposure to vacuum UV light and immersion in a Cr etchant. Surface cleaning was then conducted by immersion in sulfuric acid and rinsing with ultrapure water (18.2 MΩ cm).

### Mechanical deformation and shape characterisation of nanopillars

Nanoindentation using an ENT-2100 nanoindentator (Elionix) with a spheroid diamond tip (radius of curvature: approximately 200 µm) was performed to evaluate the mechanical strength and location-dependent mechanical deformation of the nanopillars. SQ plates with nanopillars were fixed on an automated XY stage using a Crystalbond 509 adhesive (Aremco Products). The positions required to conduct the nanoindentation process were confirmed by optical microscope observations of the micron-bars and nanopillar areas, and the XY stage was operated using a PC. Load–displacement curves were collected until a peak load of either 12 or 100 mN was recorded at a rate of 5 mN s^−1^ with an interval of 5 s at the peak load.

Sub-millimetre- and micrometre-scale individuality authentications were performed via reflection-mode optical microscopy using a BX51 microscope (Olympus) with a charge-coupled device (CCD) camera. Nanometre-scale individuality authentications were performed via FE-SEM using an S-4800 (Hitachi High-Technologies) and JSM-7800F (JEOL), as well as AFM using an S-image (Hitachi High-Tech Science) with an OMCL-AC200TS-R3 silicon cantilever (Olympus). The optical microscopy and FE-SEM images were analysed using WinRoof software (Mitani). The diameters and heights of the original and mechanically deformed nanopillars were determined from FE-SEM observations of the normal surfaces at an angle of 0° and the inclined surfaces at 70°, respectively. Pt–Pd was deposited onto the SQ surfaces for 20 s using an E-1010 ion sputtering apparatus (Hitachi High-Technologies) prior to the FE-SEM observations. A cross-sectional SQ nanopillar was prepared by exposure to a focused ion beam using a Helios NanoLab 600i FIB/SEM (FEI) and electron beam deposition of Pt into the system prior to SEM observations at a tilt angle of 52°.

## Supplementary Information

Supplementary Figures.

## Data Availability

All data generated or analysed during this study are included in this published article (and its Supplementary Information files).
