# Supplementary material for: Plastic deformation of synthetic quartz nanopillars by nanoindentation for multi-scale and multi-level security artefact metrics
Source: Sci Rep. 2021 Aug 16;11:16550. doi: 10.1038/s41598-021-95953-0 (PMC8368106; doi:10.1038/s41598-021-95953-0)
Supplement: Supplementary file 1 — Supplementary Figures. [file 41598_2021_95953_MOESM1_ESM.docx]

**Supplementary Information**

**Fig. S1.** (a) Optical microscope image of an elliptical shape consisting of plastically deformed SQ nanopillars induced by nanoindentation with a spherical diamond tip. (b) The elliptical shape highlighted in green was analysed using software.

(Microsoft Office Professional Plus 2010/Microsoft Power Point 14.0.726.5000, 32bit)

**Fig. S2.** (a) Load-displacement curves for silica nanopillars with a diameter of 200 nm and height of 100 nm measured using nanoindentation with a spherical tip. (b) Load-displacement curves with the x-axis as the normalized displacement (*h*/*h*_max_) for the curves in (a).

(Microsoft Office Professional Plus 2010/Microsoft Power Point 14.0.726.5000, 32bit/Microsoft Excel 14.0.726.5000, 32bit)

**Fig. S3.** Schematic of the UV nanoimprint lithography process for the fabrication of silica nanopillars. (a) Preparation of a spincoated UV-curable liquid film on a CrN (10 nm)/silica substrate, (b) moulding and curing via UV light exposure, (c) demoulding, (d) removal of the residual layer by reactive ion etching (RIE), (e) etching of the exposed CrN, (f) silica etching by electron cyclotron resonance reactive ion beam etching (ECR-RIBE), and (g) removal of the residual resin and CrN. (h) Pattern layout of the silica nanopillars and micrometre-scale lines.

(Microsoft Office Professional Plus 2010/Microsoft Power Point 14.0.726.5000, 32bit)
